# Social change and cohort differences in group-based arrest trajectories over the last quarter-century

**DOI:** 10.1073/pnas.2107020118

**Published:** 2021-07-26

**Authors:** Roland Neil, Robert J. Sampson, Daniel S. Nagin

**Affiliations:** ^a^Department of Sociology, Harvard University, Cambridge, MA 02138;; ^b^Heinz College, Carnegie Mellon University, Pittsburgh, PA 15213

**Keywords:** cohort, social change, offender group, arrest trajectories

## Abstract

This article draws on official criminal histories for multiple birth cohorts spanning a 17-y difference in birth year to study how social change can alter our understanding of influential theories and policies about criminal offender groups. Arrest histories are linked to comprehensive longitudinal measurement on over 1,000 individuals originally from Chicago. Using group-based trajectory modeling, we investigated the magnitude and type of cohort differences in trajectories of arrest over the period 1995 to 2020. Our results show that trajectory group membership varies strongly by birth cohort. Membership in the nonoffender group is nearly 15 percentage points higher for cohorts born in the mid-1990s as compared to those born in the 1980s; conversely, older cohorts are more likely to be members of adolescent-limited and chronic-offender groups. Large cohort differences in trajectory group membership persist after controlling for a wide-ranging set of demographic characteristics and early-life risk factors that vary by cohort and that prior research has identified as important influences on crime. Not only does the effect of social change on cohort differentiation persist, but its magnitude is comparable to—indeed larger than—differences in trajectory group membership associated with varying levels of self-control or by whether individuals grew up in high-poverty households. These results suggest that changes in the broader social environment shared by members of the same birth cohort are as powerful in shaping their trajectory group membership as classic predictors identified in prior research, a finding that carries implications for crime-control policies that rely on prediction.

## Significance

For decades, research and policy on crime have been influenced by perspectives emphasizing that the origins of offender groups, such as chronic offenders, rest on some combination of personal traits and early-life experiences. Such explanations are incomplete, however, because they do not account for social changes that differentiate successive birth cohorts over time. Our analysis shows that arrest trajectories among cohorts separated by as little as 10 y differ markedly in ways that are not explained by individual, family, or neighborhood circumstances in childhood. Analyzing cohort differences in crime is more than an overlooked strategy; it represents a class of explanation that calls for rethinking the nature of criminal offender groups and policies predicated on early-life predictors of crime alone.

The finding in the early 1970s that a small group of chronic offenders accounted for the majority of all criminal offenses (1) marked a shift in the study of crime. Reinforced by the National Research Council’s report (2), scholars increasingly turned their attention to longitudinal studies of crime, showing that not only does overall criminal involvement typically vary sharply over the life course, but that there is also substantial heterogeneity in trajectories of crime. The ability to explain why groups of people share similar patterns of crime as they age has been central to criminological theories and influential in policymaking, particularly the idea of prospectively identifying persistent offenders and for the capacity of early-childhood interventions to inhibit future crime.

Reflecting these high stakes, considerable interdisciplinary research focuses on explaining variation in trajectories of crime over the life course (3), most often with reference to individual-level characteristics and life circumstances during childhood and adolescence. While providing valuable knowledge on the importance of childhood in shaping offending trajectories (4) and on the later turning points that may deflect them (5), we argue that such perspectives are incomplete. Because societies change as individuals age, the life circumstances and social environments faced by people born at a given time—when they reach the ages at which crime is most common—may differ substantially, with potentially large consequences for trajectories of crime.

Although foundational theoretical perspectives in developmental and life-course studies of crime are consistent with this point (6, 7), the study of how social change influences trajectory groups—including which specific groups, at what life stages, and to what degree—is largely unexamined (8–10). A small body of research examines how average patterns of crime and criminal justice contact across the life course are influenced by the shared social environments of different birth cohorts (10–16). One study shows that cohorts separated by as little as one decade exhibit large variation in average patterns of arrest as they age, reflecting the differing historical contexts in which aging occurs rather than differences in the demographic composition or early-life experiences of various birth cohorts (10). Another study shows that cohort differences in the prevalence of offending may reflect changes in exposure to situations conductive to criminal behavior and contact with the criminal justice system (17).

Social changes, such as the rise of mass incarceration, rising inequality, and the decline in violence since the mid-1990s further motivate the study of cohort differences in crime rates (18–23). Cohorts coming of age in the last decade, for example, did so in a world with substantially altered patterns of policing strategies, technology use, peer conflict, and neighborhood contexts, among other changes, compared to those born one or two decades earlier.

How do we square the potential of social change to distinguish the life experiences of different birth cohorts with the study of criminal trajectory groups, which although central to scholarship on crime, has neglected cohort differentiation? The answer to this question remains unclear, as the concept of cohort has almost exclusively been used to study changes in aggregate crime rates or, in limited cases, average differences in crime over the life course (10).

## Analytic Strategy and Major Findings

We advance existing research by taking advantage of the capacity of a multicohort design to study varying offender trajectory groups across cohorts. Although longstanding methodological issues in studying age, period, and cohort are important and incorporated, our contributions are primarily substantive. Developmental trajectories of crime reflect more than just people who share similar offending patterns because of their personal traits and early-life experiences. We show that social change—as manifested through cohort differences in the distribution of offender trajectory groups—represents a key missing piece of the puzzle: developmental trajectories also reflect the power of shared social environment to shape people’s lives.

We substantiate this claim empirically by drawing on 25 y of criminal history data on multiple birth cohorts that span a 17-y period. Our study includes rich measurement on over 1,000 individuals originally from Chicago, with which we investigate the degree and nature of cohort differences in trajectories of arrest over the period 1995 to 2020. We answer two main research questions. First, how does membership in offender trajectory groups, defined by arrest, vary by cohort? Studies of trajectory groups typically employ data from people born around the same time, making cohort differences virtually impossible to observe. Second, do cohort differences in trajectory group membership reflect only that cohorts differ demographically and in their level of exposure to multiple risk factors in childhood and early adolescence? Establishing whether this is the case, or whether cohort differences reflect the dynamic influence of social environment across the life course, is crucial in order to understand what cohort differences represent and what they mean for the interpretation of trajectory groups more broadly.

Our results show that trajectory group membership varies strongly by birth cohort. Membership in the nonoffender group is nearly 15 percentage points higher for cohorts born in the 1990s as compared to those born in the 1980s; conversely, the older cohorts are much more likely to be members of adolescent-limited and persistent offender groups. Furthermore, these cohort differences in trajectory group membership persist even after controlling for a wide-ranging set of demographic characteristics and early-life risk factors that prior research has identified as important in predicting crime trajectories. Not only does the effect of social change on cohort differentiation persist, but its magnitude is found to be comparable to, indeed larger than, differences in trajectory group membership brought about by varying levels of self-control or by whether or not individuals grew up in households on welfare. These results demonstrate that the broader social environment shared by members of the same birth cohort is as important in shaping their trajectory group membership as classic predictors in criminological research, a finding that carries implications for crime control policies that rely on prediction.

While the size of the smaller, high-rate persistent offender group varies less by cohort, we do find substantial within-group variability across cohorts in the rate of arrests per offender as well as in the types of arrests of the high-rate offenders that has been overlooked by prior research. A decomposition analysis further indicates that while about two-thirds of the overall cohort difference in offending rates between the ages of 17 and 24 y are due to differing group membership, nearly one-third is due to different arrest rates within trajectory groups, which overwhelmingly reflects differences among high-rate, persistent offenders across cohorts. A major implication is that cohort differentiation through social change not only influences which trajectory group cohort members find themselves in, but also the nature of arrest patterns given membership in a trajectory group. This finding also carries implications for predictive policies, which we discuss.

## Materials and Methods

The data consist of 1,057 individuals from four different age cohorts of the Project on Human Development in Chicago Neighborhoods (PHDCN). At the PHDCN’s start in the mid-1990s, a representative sample of children belonging to seven age cohorts, starting with a newborn cohort and ranging up to age 18 y, was drawn from a representative sample of Chicago neighborhoods. About 35,000 households were first screened for eligible children, yielding an initial sample of over 6,200 children. Data on these children and their families were then collected during extensive in-home assessments, which included interviews with their primary caregiver. This initial wave was followed by two further waves of data collection over roughly 2.5-y intervals. Neighborhood data were also collected (24).

In 2011 to 2013, a random sample of those last contacted at wave 3 in the 0-, 9-, 12-, and 15-y-old age cohorts was reinterviewed, labeled “wave 4.” Parallel to waves 1 to 3, detailed information was gathered from respondents on a wide array of topics. In addition, the caretakers of cohort 0 members were asked a battery of items measuring the behavior and circumstances of their dependent as an adolescent.

Arrest records starting in 1995 were collected from the Criminal History Record Information (CHRI) in Illinois and analyzed. Wave 4 respondents were matched by name (including aliases) and date of birth with CHRI records covering the entire state of Illinois four times—in 2015, 2017, 2019, and early 2021—expanding prior work (10) by measuring the sequencing of arrests by age from 1995 through calendar year 2020. Together, these data provide rich measurement on subjects’ characteristics and the early-life family and neighborhood contexts in which development occurred, as well as arrest data over 25 y in the life course (see *SI Appendix*, Section 1 for information on attrition and out-of-state migration).

The four age cohorts included in wave 4 were observed at partially overlapping ages. Arrest data for cohort 0 are from age 10 to 25 y, for cohort 9 from age 10 to 34 y, for cohort 12 from age 17 to 37 y, and for cohort 15 from age 17 to 40 y. Arrest data for cohorts 12 and 15 prior to age 17 are excluded due to incomplete recordkeeping at the time (see *SI Appendix*, Table S1 for further information on the data structure by cohort). To improve common support across cohorts, no data past the age of 33 y are analyzed (see *SI Appendix*, Section 2 for a sensitivity check that limits maximum age to 25 y). There are, however, arrest data on all individuals from the ages of 17 to 24 y, the prime ages for arrest, and data on 58% of respondents to age 33 y. To simplify the presentation of results, in the main analyses cohorts 9, 12, and 15 are combined into what we call the “older birth” cohorts who were born between 1979 and 1988 and age cohort 0, which we call the “younger birth” cohorts because members were born between 1994 and 1996 (results are substantively unchanged when modeling the age cohorts separately).

The main dependent variable is the number of arrests at a given age. Arrests are not a pure measure of criminal offending, of course, but they constitute the primary source of information used by a long line of criminological research on criminal trajectory groups and, importantly, criminal arrests histories form the cornerstone of many prediction tools in crime control policies. Key demographic information on the sex, race/ethnicity, and immigrant generation of the subjects is measured. Subject behavioral troubles are measured with three variables capturing low self-control and impulsivity, antisocial behavior, and anxiety/depression, measured in the mid-teens. Early-life family structure and socioeconomic status are incorporated with a series of variables, all measured when subjects were children or early adolescents: household income, household size, home ownership, primary caregiver’s education level, whether the primary caregiver received TANF (public welfare assistance), whether the primary caregiver was employed, and the primary caregiver’s relationship status. Whether parents were arrested or had trouble with the law was also measured, as was whether the primary caregiver was depressed, and the number of family members that had frequent trouble with the law, employment, getting into fights, or with school discipline. Early-life neighborhood characteristics were measured with seven tract-level variables measured when respondents were 9 y of age: the poverty rate, the unemployment rate, the fraction of households that were headed by females only, the fraction of housing that was owner-occupied, the fraction of adults with a college education, as well as the fraction of residents who were black and Hispanic. Early-life neighborhood crime exposure was measured with the tract-level homicide rate around the ages of 6 to 10 y (see *SI Appendix*, Table S2 for further details on the variables used in analyses). Taken together, this set of variables represent classic childhood, family, and neighborhood predictors of crime (7, 25, 26). While cohort differences in some of these variables may themselves be the result of social change, our focus is on quantifying the role of social changes during adolescence and adulthood, motivating our strategy to adjust for early-life differences between cohorts.

Missing data are relatively rare and arrest data are complete at all ages for which a given individual was observed. Most subjects (80.5%) are missing data on fewer than 10% of the control variables, with only three subjects missing data on over 50% of them; no control variable has missing data for more than 13.2% of subjects. Ten multiple imputed datasets were created to carry out the analysis that uses variables with missing data (27), with estimates from the separate imputations combined to account for the uncertainty from imputing values (28).

Offender groups were identified using group-based trajectory modeling, a form of latent group analysis based on finite mixture modeling that uses maximum-likelihood estimation to jointly model groups’ trajectories (29, 30), in this analysis expected arrests by age and the probability of group membership. Our fundamental concept of interest is the distribution of arrests conditional on age; that is, the distribution of arrest trajectories denoted by *P*(*Y*_*i*_ | *Age*_*i*_), where the random vector *Y*_*i*_ represents individual *i*’s longitudinal sequence of arrests defining the trajectory and the vector *Age*_*i*_ represents individual *i*’s age when each of those measurements is recorded. The group-based trajectory model assumes that the population distribution of trajectories arises from a finite mixture of unknown order *J*. The likelihood for each individual *i*, conditional on the number of groups *J*, may be written as

$$P\left(Y_{i}|Age_{i}\right)=\sum_{j=1}^{J}\pi^{j}\cdot P\left(Y_{i}|Age_{i},j;\beta^{j}\right),\;$$ [1]

where *π* ^*j*^ is the probability of membership in group *j*, and the conditional distribution of *Y*_*i*_ given membership in *j* is indexed by the unknown parameter vector *β* ^*j*^, which among other things determines the shape of the group-specific trajectory. Typically, the trajectory is modeled with a polynomial function of age. For given *j*, conditional independence is assumed for the sequential realizations of the elements of *Y*_*i*_*, y*_*it*_*,* over the *T* periods of measurement. Thus, we may write

$$P\left(Y_{i}|Age_{i},j;\beta^{j}\right)=\prod_{t=i}^{T}p\left(y_{it}|age_{it},j;\beta^{j}\right),\;$$ [2]

where *p*(.) is the distribution of *y*_*it*_ conditional on membership in group *j* and the age of individual *i* at time *t*. As our dependent variable is a count variable, arrests by age, *p*(*) is specified to follow the Poisson distribution. We also note that *π* ^*j*^ is specified to follow the multinomial logit function, which allows the option of generalizing the model to condition probability of trajectory group membership on baseline covariates of *i*.

## Results

To identify the best-fitting model, Bayesian information criterion statistics were compared across models with different numbers of groups and polynomial functional forms for the age trajectories. The two best-fitting models were a three-group solution and a four-group solution, each of which specified a cubic polynomial for all of the groups’ trajectories. The size, shape, and level of two groups, which as described below, we call the “low” and “medium” trajectories, remain virtually unaltered. What changes is that the four-group solution splits the “high” group into two smaller groups that add no additional substantive insights. For parsimony, we focus on the three-group model in the main results (see *SI Appendix*, Fig. S3 for the four-group solution).

**Fig. 1.**
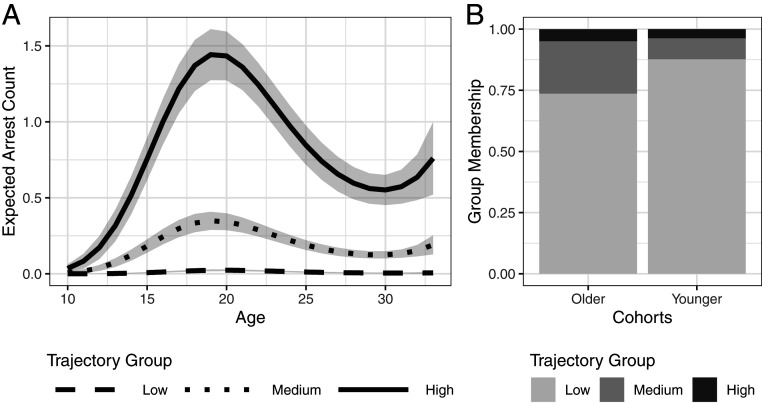
Results from the three-group model of arrest trajectories. (*A*) Age-arrest curves for the low-, medium-, and high-trajectory groups. Shaded area represents 95% confidence intervals. (*B*) Younger cohorts are more likely than older cohorts to belong to the low-trajectory group, and less likely to be in the medium- and high-trajectory groups.

Fig. 1*A* presents the expected number of arrests at a given age for each of the three trajectory groups identified. The low group has an expected arrest count of nearly zero at all ages, with a slight increase in late adolescence and early adulthood, up to a high of only 0.02. The medium group’s trajectory diverges from the low group around the age of 11 y, rising to an expected arrest count of 0.35 at age 19 before gradually falling over the next decade. The high group sharply diverges from the other two trajectories from the age of 10 y onward, rising to an expected arrest count of 1.44 at age 19 y, and falling to a low of 0.55 at age 30 y. The probabilities of membership in the low, medium, and high groups are, respectively 0.787, 0.168, and 0.045. In short, most subjects were rarely (if ever) arrested, a small fraction of the sample was arrested on average more than once per year at peak offending ages, and a sizeable minority of the sample falls between these extremes.

The slight uptick in expected arrests at age 30 y for the medium and high groups is an artifact of the cubic function that by construction has a peak and a trough. No such uptick exists in the underlying data being fit. In addition, a three-group, spline-based model of the trajectories, which otherwise looks remarkably similar to the cubic polynomial-based model, shows no such uptick (*SI Appendix*, Section 3). Furthermore, various tests of model adequacy support the fit of the three-group cubic model. The probability of trajectory group membership was nearly identical to the fraction of the sample assigned to each group based on the maximum posterior probability of membership (*SI Appendix*, Table S3). Also, the mean probability of membership in each group for subjects assigned to those groups was 0.98, 0.88, and 0.94, for the low, medium, and high groups, respectively, all of which are well above the recommended minimum level of 0.7 (30).

### Cohort Differences in Group Membership.

To measure how trajectory group membership varied between the younger cohorts and the older cohorts, the model shown in Fig. 1 was estimated in a form in which the probability of trajectory group membership, which as earlier noted follows a multinomial logit function, was specified to be conditional on cohort membership. As shown in Fig. 1*B*, the probability of membership depends substantially on cohort membership. Notably, the older cohorts are much more likely to be in the medium as opposed to the low trajectory groups than are the younger ones (odds ratio = 2.972; *P* < 0.001). For the older cohorts, probability of membership in the low and medium trajectories are, respectively, 0.74 and 0.21. In contrast, the respective probabilities for the younger cohorts are 0.88 and 0.086, a substantial absolute difference in both cases.

The cohort difference in probability of membership in the high group is less pronounced. Specifically, the odds of membership in the high group as opposed to the low group are 1.575 times higher for the older versus younger cohorts. This translates into a probability of high group membership of 0.049 for the older cohorts and of 0.037 for the younger cohorts. While not statistically significant (*P* > 0.05), the difference is in the same direction as that between the low and medium group, with the younger cohorts having lower membership in the higher-rate trajectory group. Furthermore, a 1.2 percentage point difference in expected high group membership by cohort is large given what is being estimated: people who are chronic high-rate recidivists for much of their lives. For reference, 1.2% of the US population is 4 million people; were similar effect sizes applied to that population, it would represent a very large group of people who live—or avoid—a life of frequent criminalization due to social changes.

### Adjusting for Demographics and Risk Factors.

The cohort differences reported so far may not reflect the influence of shared social environment in adolescence and adulthood on trajectories of offending but instead could reflect differences in the cohorts’ composition or in their exposure to risk factors that are determined earlier in life. This possibility is investigated with our main model specification that jointly conditions on five major demographic and risk factors: self-control, parental welfare (TANF) receipt, sex, race/ethnicity, and immigrant generation. The addition of these control variables does not explain or even attenuate the estimated cohort effect, but instead increases its magnitude. The older cohorts were estimated to have odds of membership in the medium group compared to the low group that are 4.167 times higher than the younger cohorts (*P* < 0.001). The odds of membership in the high group as opposed to the low group increase to 2.261 for the older compared to the younger cohorts, a difference that is now statistically significant at the 0.05 level (*P* = 0.027). Thus, the cohort differences in group membership do not reflect demographic differences or differences in risk factors. If anything, those factors partially mask the magnitude of the cohort effect (see *SI Appendix*, Table S4 for full output).

**Fig. 2.**
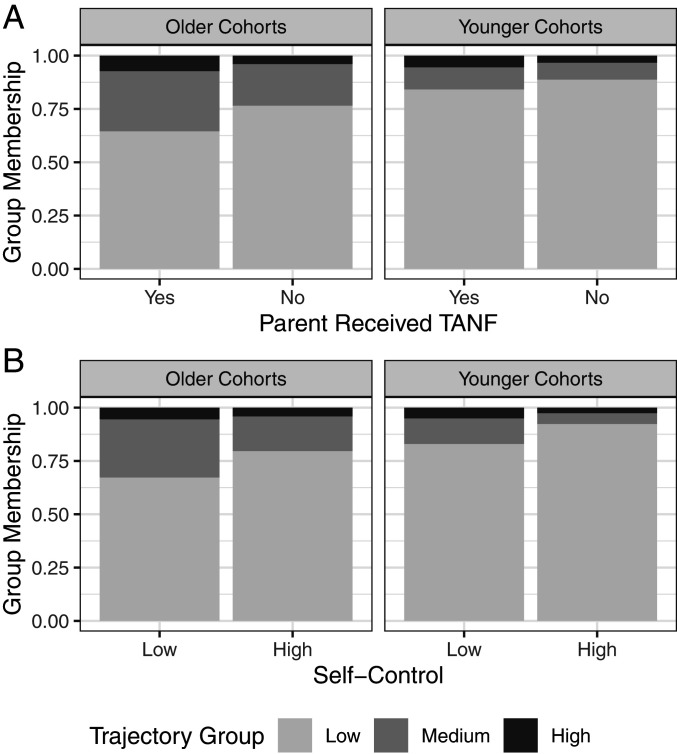
The probability of membership in the trajectory groups varies by cohort and other risk factors for crime. (*A*) Being in the older cohort reduces the probability of being in the low group by more than growing up with a parent on welfare. (*B*) Similarly, being in the older cohort reduces the probability of being in the low group by more than a 2 SD difference in self-control.

The addition of demographic and risk factors also reveals that the estimated cohort differentiation in trajectory group membership is comparable in magnitude to that of several well-established, even classic, risk factors for criminal involvement. Fig. 2*A* shows how the probability of trajectory group membership varies by cohort and by whether parents received TANF, an indicator of low socioeconomic status and economic deprivation. As expected, within each cohort children with parents on welfare are more likely to belong to the medium or high trajectory groups. Notably, however, the magnitude of the cohort effect on trajectory group membership is larger than the TANF effect. Subjects from the older cohorts have a probability of membership in the low group of 0.721 compared to 0.881 for the younger cohorts, a difference of 0.16. By comparison, having grown up in a TANF household yields a probability of membership in the low group of 0.724, as opposed to 0.797 for non-TANF households, a difference of 0.073, which is less than half the size of the cohort differential.

Fig. 2*B* presents the counterpart results comparing cohort and self-control/impulsivity levels. For purposes of comparison, we calculate the effect on trajectory group membership probabilities of a reduction from 1 SD above to 1 SD below the mean of self-control. Like with TANF, lower self-control decreases the probability of membership in the low trajectory group. Individuals 1 SD below the mean of self-control have a 0.717 probability of membership in the low group, compared to 0.843 for those 1 SD above the mean, a difference of 0.126 which is again smaller than the cohort impact of 0.16.

To further test the robustness of cohort differences to the addition of covariates measuring personal circumstances, a model that adds 19 more risk factors was estimated. We are not focused on the coefficients of the large number of risk factors, many of which are correlated. It is not surprising, for example, that the association of both TANF and self-control with trajectory group membership is attenuated in this extended model. In contrast, the estimated effect sizes of being in the older cohorts as opposed to the younger cohorts are larger: the odds ratio for membership in the medium as opposed to low group is 5.124 (*P* < 0.001) and that for membership in the high as opposed to low group is 2.638 (*P* = 0.035). Thus, being born into the earlier cohorts is associated with much greater risk of being classified as a moderate or severe offender, even after adjusting for demographic, compositional differences between cohorts, and a wide array of early-life criminogenic risk factors (see *SI Appendix*, Table S4 for full output).

### Within-Group Arrest Patterns by Cohort.

While group-based trajectory modeling identifies clusters of individuals following similar trajectories of offending, there may still be within-group variation in the rates and composition of offending. We next examined whether there are systematic differences across cohort along these two dimensions. To do so, we compared arrest patterns from the ages of 17 to 24 y, the age range for which we have complete arrest data for all subjects. This is also the age range at which arrest rates peak; as such, they constitute 59.4% of all recorded arrests.

Table 1 reports the average number of arrests per person by trajectory group and cohort. For the low and medium trajectory groups the differences between cohorts in average arrests per person is small. In contrast to these small differences, the average number of arrests within the high group differs substantially by cohort, 8.07 for the younger cohorts and 10.88 for the older cohorts (*P* = 0.065). Put differently, within the high group, the older cohorts were arrested 34.8% more often than the younger cohorts between the peak offending ages of 17 and 24 y.

**Table 1.** Arrest rate by cohort and offender group

Group	Arrest rate
Low trajectory group	
Younger cohorts	0.10
Older cohorts	0.16
Medium trajectory group	
Younger cohorts	2.52
Older cohorts	2.44
High trajectory group	
Younger cohorts	8.07
Older cohorts	10.88

Cells contain the average arrest count per person in each cohort–offender group combination, from ages 17 to 24 y.

Table 2 compares offense-specific arrests rates across cohorts and within group for four types of charges: violence, property, drugs, and other (the large majority of which are traffic offenses, ordinance violations, and warrants). Because a given arrest can carry multiple types of charges, the categorization of Table 2 is not mutually exclusive. In practice, however, arrests with some combination of drug, violence, or property charges are rare in these data. Again, we see that for the low and medium groups there are small differences by offense type between the cohorts and none are statistically significant.

**Table 2.** Offense-specific arrest rate by cohort and offender group

Group	Offense type			
	Drugs	Violence	Property	Other
Low trajectory group				
Younger cohorts	0.03	0.02	0.02	0.05
Older cohorts	0.04	0.03	0.04	0.08
Medium trajectory group				
Younger cohorts	0.42	0.68	0.55	1.29
Older cohorts	0.66	0.49	0.61	0.99
High trajectory group				
Younger cohorts	1.27	1.67	2.27	4.60
Older cohorts	3.66	1.53	2.38	4.81

Cells contain the average offense-specific arrest count per person in each cohort–offender group combination, from ages 17 to 24 y.

Turning to the high group, arrest rates across cohorts are similar except for drug arrests, for which the arrest rate is 1.27 for the younger cohorts compared to 3.66 for the older cohorts, a difference that is statistically significant (*P* < 0.01). This large difference in drug arrest rates between cohort for the high group almost fully explains the differences in total arrests for the high group reported in Table 1. However, refitting the trajectory group models, while leaving out arrests that only had drug charges but otherwise using the same specifications and controls, leads to very similar estimates and the same substantive conclusion as when including drug arrests. Thus, cohort differences in drug arrest rates do not explain away cohort differences in trajectory group membership; they are distinct forms of cohort differences in arrests. In addition, there is no significant interaction between race and cohort in the prediction of trajectory group membership. This further indicates that cohort differences in trajectory groups do not only reflect changes in the war on drugs, to which African Americans in the older cohorts would have been most exposed (31, 32).

### Decomposition of Cohort Differences in Arrests.

A decomposition analysis was conducted to partition the cohort difference in overall arrest rates between differences in trajectory group membership and differences in within-trajectory group rates of arrest. These portions can be estimated by counterfactually treating each cohort as if it had either the same trajectory group membership or within-group offending rates as the other cohort and comparing the resulting offense rates to the observed cohort offense rates.

The actual arrest rate per person (from the ages 17 of 24 y) for the younger cohorts is 0.614 compared to 1.156 for the older cohorts, a gap of 0.542. If the trajectory groups of the younger cohorts had the arrest rates of the older cohorts by trajectory groups, their arrest rate would have been 0.774. That is 26% higher than it actually was. If the younger cohorts belonged to the trajectory groups in the same proportions as the older cohorts, but holding their actual arrest rates within each trajectory group constant at the observed values, then the arrest rate for the younger cohorts would have been 0.995 or 62% higher than it actually was. Thus, both differing trajectory group sizes and within-group arrest rates account for differences in the average rate of arrests between cohorts. Notably, if trajectory membership of the younger cohorts had remained at observed levels and within the low and medium trajectories arrest rates had similarly remained unchanged, with only the arrest rate of the younger cohorts in the high group increasing to that of the older cohorts’ arrest rate for that group, the younger cohorts’ rate would have been 0.725, or 18.2% higher than it was. This calculation confirms that the difference in the arrest rates between cohort in the small high trajectory group was an important factor in driving the overall differences in arrest rates between cohorts.

## Discussion

Which people will go on to commit the most crimes or be arrested the most, and the rate at which they do so, cannot be known based on individual background, personal characteristics, early-life risk factors, and age alone. This result was obtained because membership in trajectory groups, as well as the nature of crimes among individuals seemingly in the same group, are both strongly influenced by the larger social environment, which itself changes over time. That is, our results indicate that arrest trajectories are outcomes that are responsive to changing historical contexts, not just to early-life circumstances or individual characteristics determined at birth or in early childhood.

These findings are consistent with major developmental and life-course theories of crime, which incorporate environmental influences, including historical context (6, 33). Crucially, however, prior work is incomplete, reflecting predominant theoretical emphases and the paucity of multicohort designs. In particular, existing research typically conveys trajectory groups as groupings of fundamentally similar people in terms of individual-, family-, or neighborhood-level characteristics. Our results reveal that similarity also extends to shared external or contextual environments that influence trajectories of arrest, above and beyond early-life characteristics. This is not a small detail, given the magnitude of estimated cohort effects on trajectory group sizes and the composition of their arrests.

The implication is that greater attention in the study of crime should be given to changing historical contexts. For example, the original theoretical account of adolescent-limited offenders’ behavior portrayed it as shaped by historical context (6, 8); this has gone largely overlooked, although our results offer strong support for the idea, and extend it by quantifying how much changes in historical context matter. Furthermore, while both developmental and cohort-based research typically emphasize the effects of childhood environment, our findings indicate that social context continues to play a major role in shaping developmental trajectories past childhood and into early adulthood, and itself changes alongside the development of individuals.

Our findings reveal cohort differences in every trajectory group studied. While chronic offenders likely face elevated rates of early-life adversities that set them along high-rate offending trajectories, for social or psychopathological reasons, their development is still influenced to a large degree by what has yet to happen in the world around them. It follows that a fuller understanding of developmental trajectory groups demands accounting for cohort differences in social environments throughout the life course.

Future research is needed to disentangle the societal-level changes that may have contributed to cohort differences in trajectory groups. For example, during this period Chicago’s neighborhoods were undergoing an array of changes that may have enhanced social control while reducing opportunities for crime, such as revitalization from immigration, the mobilization of local organizations against violence, and the replacement of segregated public housing with mixed-income developments. In addition, the lives of youth in Chicago and beyond were transformed by the rise of novel technologies, ranging from smart phones to social media, as time spent engaging in unstructured socialization decreased (17, 34). Moreover, arrest patterns reflect not only changing criminal behaviors but also changes in policing. Drug policing was becoming notably less aggressive in Chicago over this period (10), although our results indicate that this alone cannot explain cohort differences in trajectory group membership. Because trajectory groups—and arrest patterns more broadly—reflect cohort contextual effects to such a large extent, it is important that future research seek to establish which aspects of the shared social environment are most responsible for producing cohort differentiation. As the COVID-19 pandemic and recent increases in violent crime drive home, social change is not just history, but an ongoing, powerful, and often unexpected process. There is no inherent directionality to cohort contextual effects, meaning that our capacity to understand changing arrest and crime patterns requires being attentive to novel social changes and measuring how they impact the developmental patterns of contemporary cohorts.

Our findings also carry policy implications. For one, they suggest there are real limits to how well offender groups and offending patterns can be prospectively identified. Predicting the future risk of offending is a core component of many crime-control policies, including pervasive attempts to identify at-risk youth, as well as the recent emphasis on using machine-learning algorithms in the criminal justice system (35). Yet algorithms trained on past data may be prone to make inaccurate predictions because of the fact that they do not incorporate the influence of cohort and historical period. Our findings imply that even an algorithm trained on a cohort born just 10 y before those for whom predictions are being made may misestimate the risks posed by individuals in the later cohort.

As illustrated by the controversy surrounding the COMPAS risk assessment instrument (36), much research on algorithmic bias in the criminal justice system has focused on biases related to race and gender and the challenges of correcting for such bias (35, 37, 38). Our research suggests that biases may extend to entire cohorts. That we found no significant interactions between cohort and two key foci of research on algorithmic bias—race and gender—suggests that cohort is a distinct source of bias. We therefore recommend that future research give priority to identifying both the type and magnitude of cohort-based algorithmic biases that may be present. Whereas the COMPAS instrument may be well-calibrated despite producing racial differences in prediction error rates (37), our results imply that algorithms trained on prior cohorts may produce poorly calibrated predictions for subsequent cohorts, and possibly cohort differences in prediction error rates.

An important question is the extent to which gaining information on individuals’ prior criminal history records—the central component of criminal justice risk instruments—can correct for cohort-based prediction biases. Future research should distinguish prediction biases related to the level of offending from an individual’s relative ranking among cohort members. Both types of biases may be present, but the former is most relevant to criminal justice policy because the social cost of crime relates to an individual’s level of offending, not from their relative ranking.

Finally, our findings suggest the value of broadening crime-prevention policy, even as it relates to the highest-rate offender group, from a narrow focus on identifying individuals whose future offending patterns may be high to enhancing opportunities and structures in the larger social environment. We urge this because an individual’s life is determined not only by who they are or what they have done, but also by the changing social circumstances of when they are.

## Supplementary Material

Supplementary File

## Data Availability

Restricted-access data and replication code are available at https://doi.org/10.7910/DVN/Q7EPRI.
