# Supplementary material for: Social change and cohort differences in group-based arrest trajectories over the last quarter-century
Source: Proc Natl Acad Sci U S A. 2021 Jul 26;118(31):e2107020118. doi: 10.1073/pnas.2107020118 (PMC8346896; doi:10.1073/pnas.2107020118)
Supplement: Supplementary File [file pnas.2107020118.sapp.pdf]

PNAS
www.pnas.org

Supplementary Information Appendix for:

Social Change and Cohort Differences in Group-Based Arrest Trajectories over the Last Quarter-Century

Roland Neil[a], Robert J. Sampson[a,1], and Daniel S. Nagin[b]

[a]Department of Sociology, Harvard University, Cambridge, MA 02138;

[b]Heinz College, Carnegie Mellon University, Pittsburgh, PA 15213

[1]To whom correspondence should be addressed. E-mail: rsampson@wjh.harvard.edu.

This PDF file includes:

Main Text
Figures S1 to S3
Tables S1 to S4

Section 1: Attrition, Survey Design, and Migration out of Illinois

Of the original PHDCN sample, which was recruited with an overall 75% participation rate at wave 1 (ranging from 72-76% for the cohorts analyzed in this paper), the interview response rate at wave 3 was 78% of eligible participants, both relatively high for an urban sample. The primary caregiver response rates for the younger and older cohorts varied between 77-79% at wave 3. Of the PHDCN participants followed through wave 4 more than a decade later, 63% of eligible respondents took part, a number which varies from 61 to 67% by cohort status, with the youngest cohorts having the highest participation rate. Again, while not low for a contemporary urban sample and cohort differences are small, differential response rates over time do leave open the possibility of nonrandom attrition that could affect the results.

To address this potential, we carried out analyses using sampling weights to account for attrition and features of the survey design. The sampling weight is designed to adjust for the original stratification of the PHDCN by neighborhood SES and racial composition, along with the age cohort selection and a post-stratification of population weights to estimates of the age, gender, and race/ethnicity distribution of children in Chicago in 1995. Weights designed to adjust for attrition account for a wide range of information, including individual- and household-level measures of socioeconomic status and family composition, as well as neighborhood-level measures of demographic composition and social structure. The arrest history of the participants through wave 3 is included as a predictor to account for potential bias in follow-up. Although criminalized men are often among hardest to reach, the probability of follow-up was only marginally different among arrested and non-arrested youth.

The resulting sampling weight was used in a group-based trajectory model that was otherwise identical to the full model—that with cohort and 24 control variables. In the unweighted version, as reported in the main text, the odds ratio of the older cohorts compared

to the younger cohorts of being in the medium versus low group is 5.12 ($p < 0.01$), and the odds ratio is 2.64 ($p < 0.05$) for the high versus low group. Incorporating the weight yields analogous odds ratios of 4.27 ($p < 0.01$) and 4.27 ($p < 0.05$), respectively. While applying survey design and attrition weights does change the cohort estimates, as expected, they remain large, significant, and in the same direction. The main results are thus robust to weighting.

Data only exist on arrests that occurred across the state of Illinois. Yet some respondents moved out of Illinois, meaning that their arrests may be undercounted, which could be problematic if they were correlated with cohort status. Fortunately, the vast majority of respondents were still living in Illinois as of wave 4, and this prevalence does not vary significantly or meaningfully by cohort status (87 to 88%). Further, many of those who left Illinois by wave 4 did so past the peak ages of arrest, meaning that most arrests occurred while respondents lived in the state. Some people also moved out only to move back at a later point. To check the sensitivity of findings to migration status, the full model was refit leaving out those who did not reside in Illinois at waves 2, 3, and 4 (16 respondents). The odds ratio for membership in the medium versus low group for the older versus younger cohorts was 4.946 ($p < 0.001$), and 2.558 ($p = 0.042$) for the high vs low group. These estimates compare, respectively, to odds ratios of 5.124 ($p < 0.001$) and 2.638 ($p = 0.035$) for the model using the entire sample, as reported in the main text. The main results are thus robust to adjusting for out of state location at all follow-up waves.

Section 2: Sensitivity to Maximum Age Modelled and the Influence of Incarceration

Fig. S1 presents results from a model that is analogous to that used to produce Fig. 1 but which limits the maximum age of arrest data used to 25 as opposed to 33. With this model there is common support across the older and younger cohort groupings, from ages 10 to 25. The same

patterns of cohort differences in arrest trajectories can be seen in Fig. S1 as in Fig. 1, a finding that carries two implications.

For one, it means that cohort differences in trajectory membership are not driven by the fact that in the model used in the main results there is more arrest data (going past the age of 25) for the older cohorts than younger cohorts.

Crucially, this finding also indicates that results are not driven by cohort differences in time spent off the street, or more precisely, by differences in incapacitation due to incarceration. Only 58 of the 1,057 (5.5%) have been sentenced to jail or prison, but among them the average age of first incarceration is 23.8 years old. Thus, by limiting analysis to a maximum age of 25, most of the incarceration time that the sample would go on to experience is not included and its influence on the trajectories of Fig. S1 is likely minimal.

Incarceration could still influence trajectory membership somewhat. Across cohorts, no one in the low group was incarcerated by age 25 (or to date); their group membership is unaffected by incarceration. About 50% of the high group has been incarcerated by age 25, a number that holds across cohorts. Even if incarcerated individuals on the high group were not incarcerated, they very likely would have ended up in the high group given more street time, which is to say incarceration doesn't affect their group membership. However, the medium group does show variation by cohort. Only one younger cohort medium group member (of 31) has been incarcerated for a year or more (and one other for 8 days), as opposed to 8 of 134 (5.5%) of the older cohort members. Some of these incarcerated people may have been arrested more absent their long incarceration stints, which may have put some into the high group. This means that the cohort differences in high rates of latent criminal propensity may be larger than the observed cohort differences in high arrest trajectory group membership. These

results also identify the changes to mass incarceration as a plausible macrosocial change that influences offender group membership differences by cohort and historical period.

Section 3: The Functional Form of the Age-Arrest Trajectories

In order to examine how well the cubic polynomial specifications used in main analyses fit the data, two additional checks were performed.

First, the cubic curves track quite closely the means of the actual data that are being fit. These are weighted means, in which each individual's posterior probability of membership in each group is used as a weight so that they contribute information to each curve in proportion to how confident the model is that they belong to that trajectory group. These weighted means are presented as points in Fig. S2 below. Notably, there is no uptick in the points towards the right side of the graph.

Second, the data were fit using splines as a semi-parametric alternative to the cubic specification. Specifically, one Poisson model was fit for each trajectory group, with each model using the entire dataset of 1,057 respondents, but weighting individuals by their posterior probability of membership in that group. The relationship between age and arrests for each curve was specified with natural splines using 3 degrees of freedom. The group-specific results are plotted as lines in Fig. S2. The close correspondence between the lines and points indicates the spline models fit the underlying data well. Crucially, these lines also look very similar to Fig. 1A. The exception is at the right tail, where the spline models show no uptick in expected arrest counts, consistent with the underlying data being fit.

Table S1. Arrest Data Range and Sample Size by Age Cohort

Cohort [a]	Respondents (N)	Person-Year Observations	Age Range for Arrest Data	Number of Respondents Arrested (%)	Total Number of Arrests (avg. per arrestee)
0 (Birth)	378	5,775	10-25	86 (22.8)	320 (3.7)
9	226	5,352	10-33	106 (46.9)	463 (4.4)
12	236	4,012	17-33	95 (40.3)	418 (4.4)
15	217	3,689	17-33	88 (40.6)	406 (4.7)
**TOTAL**	**1,057**	**18,828**	**10-33**	**375 (35.5)**	**1,607 (4.3)**

[a] Cohorts are defined by subjects' age at the study's start in 1995.

Tables S2. Description and Data Sources for Variables Used in Analyses

Variable	Description	Data Source
Arrest	Outcome. Number of times SP was arrested at a given age.	CHRI
Age	Age of SP measured in one-year intervals.	W1
Older Cohort	Whether SP was born between 1979-88 (baseline = born in 1990s).	W1
Race/Ethnicity	Factor: whether SP is White, Black, Hispanic, or Other.	W1
Sex	Whether SP is male (baseline = female).	W1
Immigrant Generation	Factor: whether PC is 1st, 2nd, or higher generation immigrant.	W1
Welfare Receipt	Whether PC was on TANF.[a]	W1, W3
Low Self-control/Impulsivity	Standardized scale, parent report from CBCL checklist.[b]	W1-W4
Anxiety/Depression	Standardized scale, parent report from CBCL checklist.[b]	W1-W4
Aggression/Antisocial Behavior	Standardized scale, parent report from CBCL checklist.[b]	W1-W4
Parental Employment	Whether PC was working.[a]	W1, W3
Low Parental Education	Whether PC's max educational attainment was HS or less.[a]	W1, W3
Household Income	Factor: three levels ranging from < $20,000 to > $50,000.[a]	W1, W3
Parental Relationship Status	Factor: whether PC was single, cohabitating, or married.[a]	W1, W3
Homeownership	Whether PC owns own home.[a]	W1, W3
Household Size	Number of people residing in SP's household.	W1
Parental Depression	Whether PC is depressed.	W1
Parent Arrest	Whether a parent had trouble with police or been arrested.	W1
Family Institutional Troubles	No. family members that have had frequent trouble with the law, holding a job, getting into fights, or school discipline.	W1
Proportion Black	Tract-level fraction of Black residents when SP was age 9.[c]	CN
Proportion Hispanic	Tract-level fraction of Hispanic residents when SP was 9.[c]	CN
Concentrated Poverty	Tract-level poverty rate when SP was age 9.[c]	CN
Unemployment Rate	Tract-level unemployment rate when SP was age 9.[c]	CN
Female-Headed Households	Tract-level fraction of households headed by a female only when SP was age 9.[c]	CN
Owner-Occupied Rate	Tract-level fraction of households that were owner-occupied when SP was age 9.[c]	CN
College Education	Tract-level fraction of adults who were college-educated when SP was age 9.[c]	CN
Homicide Rate	Tract-level rate of homicides per 100,000 residents when SP was between about ages 6 and 10.[c]	CPD

Notes: SP= study participant (respondent); PC= primary caregiver (usually parent); CHRI= Criminal History Record Information (State of Illinois); W1= PHDCN Longitudinal Cohort Study (LCS) wave 1; W3 = PHDCN LCS wave 3, W4= PHDCN LCS wave 4; CN = Census; CPD = Chicago Police Department.

[a] Multi-wave variables are measured at W1 for cohorts 9, 12, and 15, and at W3 for cohort 0, so that they are measured at ages that are as similar as possible across cohorts given the data structure.

[b] Measured in mid-teens, at average age 15 across cohorts.

[c] Address histories by year are used to measure neighborhood census variables at around age 9 for each cohort. Homicide rates were only available for Chicago when the SP was between about ages 6 and 10.

Table S3. Comparison of Proportion Assigned to Posterior Probabilities of Membership by Group Indicates a Good Model Fit

Trajectory Group	Proportion of Sample Assigned to Group	Posterior Probability of Membership in Group
Low	0.7881	0.7867
Medium	0.1675	0.1684
High	0.0445	0.0448

Table S4. Regression Output for the Group-Based Trajectory Models of Arrest

	Model 1	Model 2	Model 3	Model 4
Intercept - Low Group	-36.792 (7.9)	-37.368 (7.437)	-36.076 (7.25)	-35.881 (8.17)
Linear - Low Group	4.244 (1.085)	4.375 (1.03)	4.202 (1.005)	4.181 (1.135)
Quadratic - Low Group	-0.176 (0.048)	-0.184 (0.046)	-0.177 (0.045)	-0.177 (0.051)
Cubic - Low Group	0.002 (0.001)	0.002 (0.001)	0.002 (0.001)	0.002 (0.001)
Intercept - Medium Group	-24.954 (2.82)	-24.685 (2.79)	-24.665 (2.877)	-24.015 (3.314)
Linear - Medium Group	3.18 (0.391)	3.129 (0.386)	3.112 (0.395)	3.005 (0.457)
Quadratic - Medium Group	-0.137 (0.018)	-0.134 (0.017)	-0.133 (0.018)	-0.129 (0.02)
Cubic - Medium Group	0.002 (0)	0.002 (0)	0.002 (0)	0.002 (0)
Intercept - High Group	-21.906 (2.528)	-22.126 (2.55)	-22.658 (2.466)	-22.313 (2.565)
Linear - High Group	2.934 (0.351)	2.963 (0.354)	3.035 (0.341)	2.986 (0.358)
Quadratic - High Group	-0.125 (0.016)	-0.126 (0.016)	-0.129 (0.015)	-0.129 (0.016)
Cubic - High Group	0.002 (0)	0.002 (0)	0.002 (0)	0.002 (0)
Constant - Medium Group	-2.323 (0.224)	-4.134 (0.447)	-4.078 (1.239)	-4.456 (1.34)
Older Cohorts - Medium Group	1.089 (0.247)	1.427 (0.278)	1.634 (0.331)	1.689 (0.366)
Race/Ethnicity: Black - Medium Group		1.32 (0.35)	0.942 (0.55)	1.065 (0.605)
Race/Ethnicity: Hispanic - Medium Group		1.001 (0.433)	0.438 (0.539)	0.641 (0.607)
Race/Ethnicity: Other - Medium Group		1.059 (0.608)	1.207 (0.681)	1.639 (0.729)
Sex: Male - Medium Group		1.458 (0.233)	1.638 (0.259)	1.591 (0.282)
1st Gen Imm. - Medium Group		-0.86 (0.386)	-1.053 (0.439)	-1.074 (0.485)
2nd Gen Imm. - Medium Group		-1.131 (0.546)	-1.37 (0.604)	-1.668 (0.683)
Low Self Control - Medium Group		0.48 (0.1)	0.442 (0.171)	0.474 (0.186)
TANF - Medium Group		0.461 (0.242)	-0.482 (0.319)	-0.379 (0.34)
Low Education - Medium Group			0.338 (0.283)	0.376 (0.311)
Parental Employment - Medium Group			-0.238 (0.265)	-0.293 (0.289)
Single Parent - Medium Group			0.035 (0.304)	0.261 (0.319)

	Model 1	Model 2	Model 3	Model 4
Cohabitating Parent - Medium Group			0.057 (0.387)	0.043 (0.427)
Homeownership - Medium Group			-0.757 (0.283)	-0.823 (0.317)
Income: <$20k - Medium Group			0.87 (0.47)	0.847 (0.506)
Income: >$50k - Medium Group			0.234 (0.398)	0.175 (0.437)
Household Size - Medium Group			0.087 (0.057)	0.094 (0.065)
Parental Depression - Medium Group			0.169 (0.409)	-0.457 (0.505)
Parental Arrest - Medium Group			0.441 (0.327)	0.618 (0.351)
Family Inst. Troubles - Medium Group			-0.022 (0.181)	-0.021 (0.197)
Aggression - Medium Group			0.535 (0.155)	0.393 (0.178)
Anxiety/Depression - Medium Group			-0.445 (0.173)	-0.362 (0.192)
Prop. Black - Medium Group			0.825 (0.899)	0.897 (0.956)
Prop. Hispanic - Medium Group			0.894 (0.846)	0.894 (0.923)
Female-Headed HH - Medium Group			-0.211 (1.515)	-0.111 (1.7)
Unemployment Rate - Medium Group			-4.532 (3.879)	-5.047 (4.414)
Concentrated Poverty - Medium Group			0.079 (2.261)	0.375 (2.543)
Owner-Occupied Rate - Medium Group			-0.598 (1.024)	-0.419 (1.127)
College Education - Medium Group			-0.231 (1.362)	-0.041 (1.506)
Homicide Rate - Medium Group			-0.002 (0.003)	-0.002 (0.003)
Constant - High Group	-3.162 (0.299)	-6.356 (0.824)	-6.898 (2.074)	-6.01 (2.066)
Older Cohorts - High Group	0.454 (0.346)	0.816 (0.369)	0.97 (0.46)	0.751 (0.467)
Race/Ethnicity: Black - High Group		1.463 (0.581)	0.386 (0.912)	0.06 (0.873)
Race/Ethnicity: Hispanic - High Group		1.291 (0.696)	0.11 (0.884)	-0.042 (0.859)
Race/Ethnicity: Other - High Group		1.118 (0.947)	0.332 (1.187)	0.396 (1.159)
Sex: Male - High Group		2.958 (0.564)	3.361 (0.599)	3.036 (0.549)
1st Gen Imm. - High Group		-1.211 (0.57)	-1.143 (0.669)	-1.251 (0.677)
2nd Gen Imm. - High Group		-2.321 (1.363)	-2.37 (1.246)	-2.48 (1.216)

	Model 1	Model 2	Model 3	Model 4
Low Self Control - High Group		0.408 (0.166)	0.08 (0.266)	0.066 (0.267)
TANF - High Group		0.758 (0.364)	0.442 (0.485)	0.367 (0.491)
Low Education - High Group			1.383 (0.457)	1.324 (0.459)
Parental Employment - High Group			0.101 (0.451)	-0.14 (0.453)
Single Parent - High Group			1.149 (0.509)	1.002 (0.495)
Cohabitating Parent - High Group			1.158 (0.557)	1.225 (0.553)
Homeownership - High Group			0.256 (0.451)	0.12 (0.458)
Income: <$20k - High Group			-0.764 (0.796)	-0.54 (0.81)
Income: >$50k - High Group			-0.285 (0.67)	-0.202 (0.675)
Household Size - High Group			0.057 (0.091)	0.043 (0.097)
Parental Depression - High Group			0.956 (0.532)	0.931 (0.515)
Parental Arrest - High Group			0.589 (0.507)	0.653 (0.505)
Family Inst. Troubles - High Group			0.364 (0.211)	0.418 (0.195)
Aggression - High Group			1.09 (0.24)	1.063 (0.244)
Anxiety/Depression - High Group			-0.628 (0.293)	-0.628 (0.295)
Prop Black - High Group			0.834 (1.509)	0.972 (1.547)
Prop Hispanic - High Group			1.051 (1.384)	0.982 (1.439)
Female-Headed HH - High Group			-0.793 (2.315)	-0.393 (2.258)
Unemployment Rate - High Group			0.725 (5.645)	0.35 (5.669)
Concentrated Poverty - High Group			-0.436 (3.522)	-0.517 (3.564)
Owner-Occupied Rate- High Group			-2.808 (1.588)	-2.88 (1.626)
College Education - High Group			-0.512 (2.393)	-0.615 (2.44)
Homicide Rate - High Group			0.001 (0.003)	0.001 (0.003)

Note: Coefficients are in log odds form, standard errors are in parentheses. Model 1 = baseline cohort risk model; Model 2 = with 5 control variables added; Model 3 = with 19 further control variables; Model 4= same as Model 3 but removing arrests that only had drug charges from the outcome variable. N = 1,057.

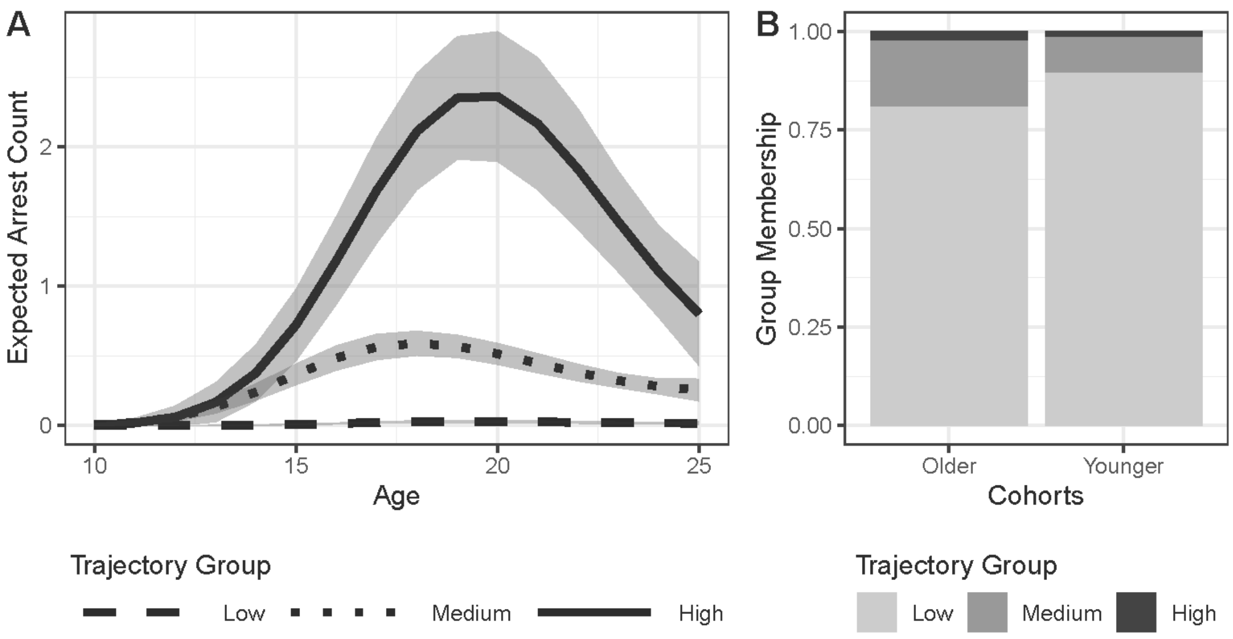

Fig. S1. Cohort differences in group membership exist in a model that limits max age to 25. (A) Expected arrest count by age for the Low, Medium, and High offender groups, shaded by 95% confidence intervals. (B) The probability of membership in each trajectory group differs by cohort.

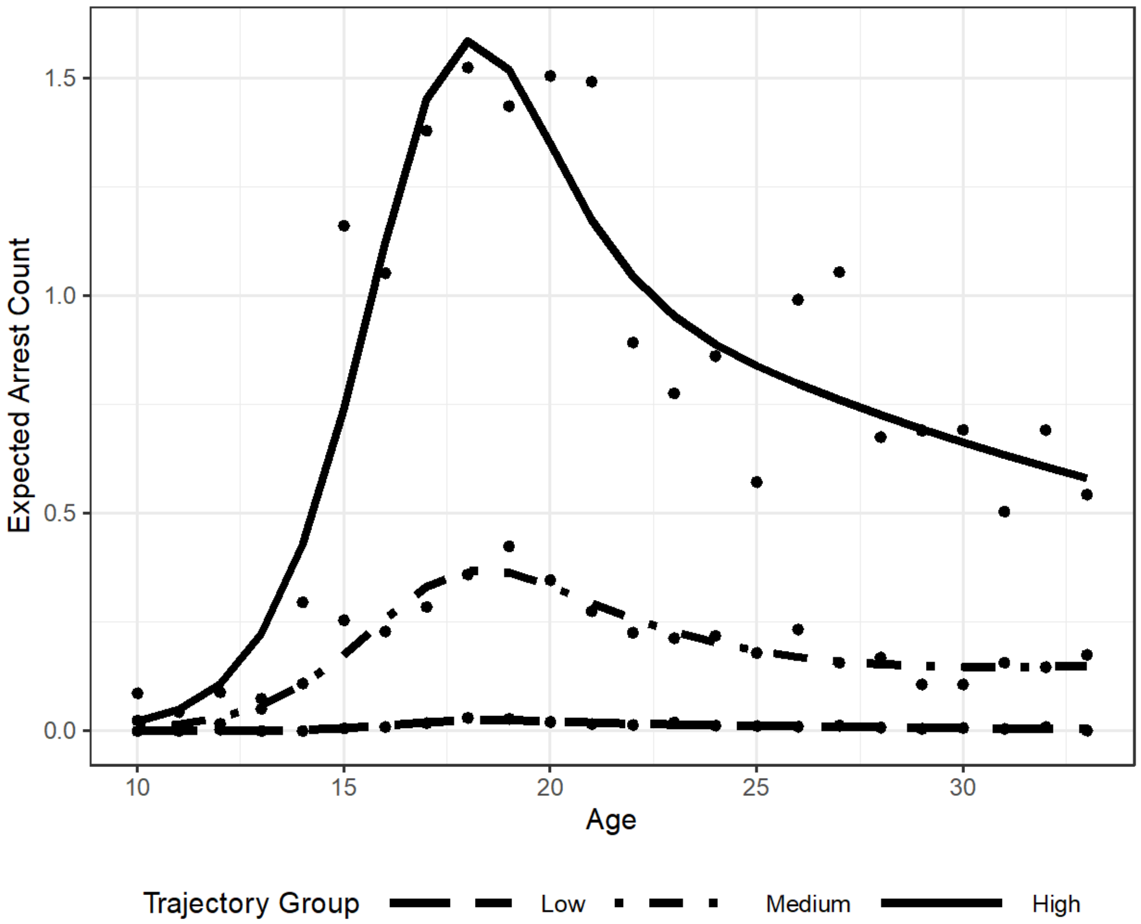

Fig. S2. Arrest trajectories as estimated with natural splines. The points represent the mean arrest rate by age and trajectory group in the underlying data. Neither the splines nor these points show an uptick on the right side of the plot. Otherwise, these results quite closely resemble Fig. 1A, indicating that model fits the data well.

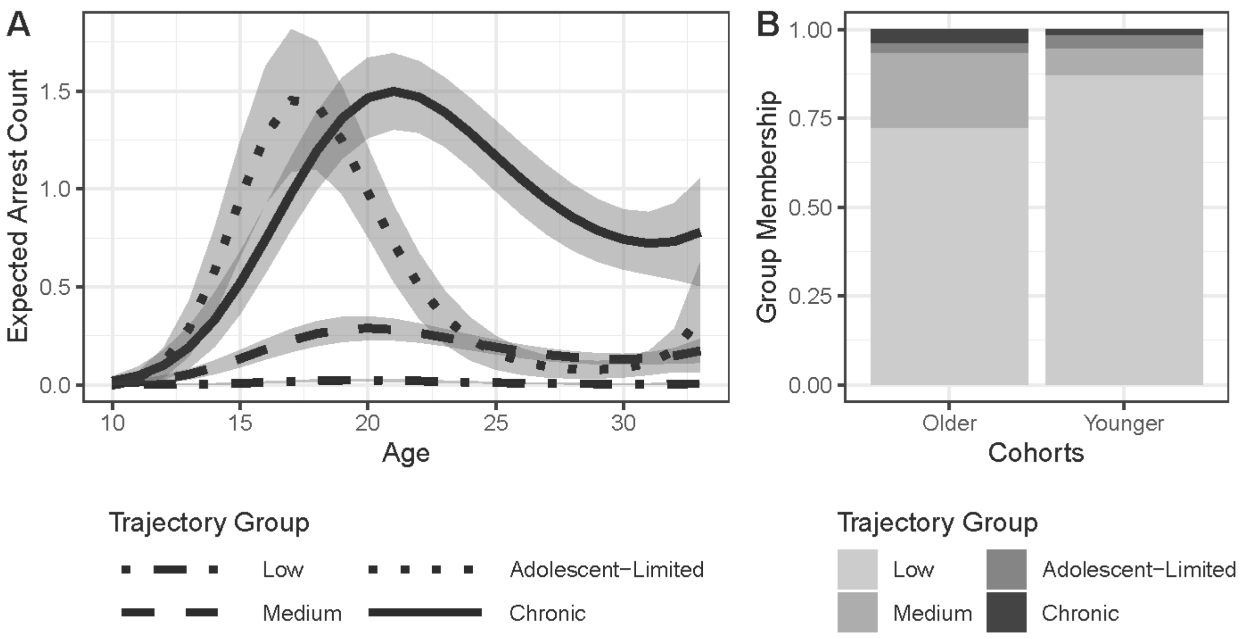

Fig. S3. Results from the 4-group model of arrest trajectories. (A) The 4-group model, shaded by 95% confidence intervals, is similar to the 3-group model, but the highest-rate offender group is now broken into two smaller groups. (B) Cohort differences in group membership persist, but the younger cohorts are more likely to be members of the new, adolescent-limited group.
